# Assessment of mitral valve geometry in nonvalvular atrial fibrillation patients with or without ventricular dysfunction: insights from high volume rate three-dimensional transesophageal echocardiography

**DOI:** 10.1007/s10554-023-02940-9

**Published:** 2023-09-04

**Authors:** Wenjuan Bai, Ying Chen, Yue Zhong, Ling Deng, Dayan Li, Wei Zhu, Li Rao

**Affiliations:** Department of Cardiology, West China Hospital of Sichuan University, 37 Guo Xue Xiang, Chengdu, Sichuan 610041 China

**Keywords:** Atrial functional mitral regurgitation, Mitral valve, Nonvalvular atrial fibrillation, Transesophageal echocardiography

## Abstract

Meticulous understanding of the mechanisms underpinning mitral regurgitation in atrial fibrillation (AF) patients is crucial to optimize therapeutic strategies. The morphologic characteristics of mitral valves in atrial functional mitral regurgitation (FMR) patients with and without left ventricular (LV) dysfunction were evaluated by high volume rate (HVR) three-dimensional transesophageal echocardiography (3D-TEE). In our study, 68 of 265 AF patients who underwent 3D-TEE were selected, including 36 patients with AF, FMR, and preserved LV function (AFMR group) and 32 patients with AF, FMR, and LV dysfunction (VFMR group). In addition, 36 fever patients without heart disease were included in the control group. Group comparisons were performed by one-way analysis of variance for continuous variables. The left atrium (LA) was enlarged in the AFMR and VFMR groups compared with the control group. The mitral annulus (MA) in the AFMR group was enlarged and flattened compared with the control group and was smaller than in the VFMR group. The annulus area fraction was significantly diminished in the AFMR and VFMR groups, indicative of reduced MA contractility. The posterior mitral leaflet (PML) angle was smallest in the AFMR group and largest in the control group, whereas the distal anterior mitral leaflet angle did not significantly differ among the three groups. LA remodeling causes expansion of the MA and reduced MA contractility, disruption of the annular saddle shape, and atriogenic PML tethering. Comparison of atrial FMR patients with and without LV dysfunction indicates that atriogenic PML tethering is an important factor that aggravates FMR. HVR 3D-TEE improves the 3D temporal resolution greatly.

## Introduction

Atrial fibrillation (AF) is a common arrhythmia whose prevalence increases with advancing age. [1, 2] AF induces left atrial (LA) remodeling, and functional mitral regurgitation (FMR) can occur as a result of LA dilatation in patients with AF, despite preservation of the left ventricular (LV) ejection fraction (LVEF). [3] This is termed atrial FMR, which is linked to a worse outcome. [4, 5] With the rapid development of transcatheter solutions, there is a growing need to understand the mechanism underlying atrial mitral regurgitation (MR) in order to determine appropriate management solutions.

In previous studies, mitral valve (MV) volume data were acquired by three-dimensional (3D) zoom or one-beat full-volume transesophageal echocardiography (TEE), which has low temporal resolution, usually 6–16 volumes per second, and the low frame rate affected the measurement accuracy. [6, 7] Several studies acquired MV volume data using four heartbeat stitching to improve temporal resolution, and this was only applied in patients with sinus rhythm. Recently, high volume rate (HVR) 3D-TEE was developed, in which an ultrasound system automatically recognizes and selects the synthetic volume modality. This technology uses a more refined algorithm that maintains both temporal resolution and avoids the stitching artifact. HVR 3D-TEE can also be applied to acquire volumes in arrhythmia subjects. Our study aimed to investigate the geometrical differences of the MV between AF patients with and without LV dysfunction.

## Methods

### Patients

**Fig. 1:**
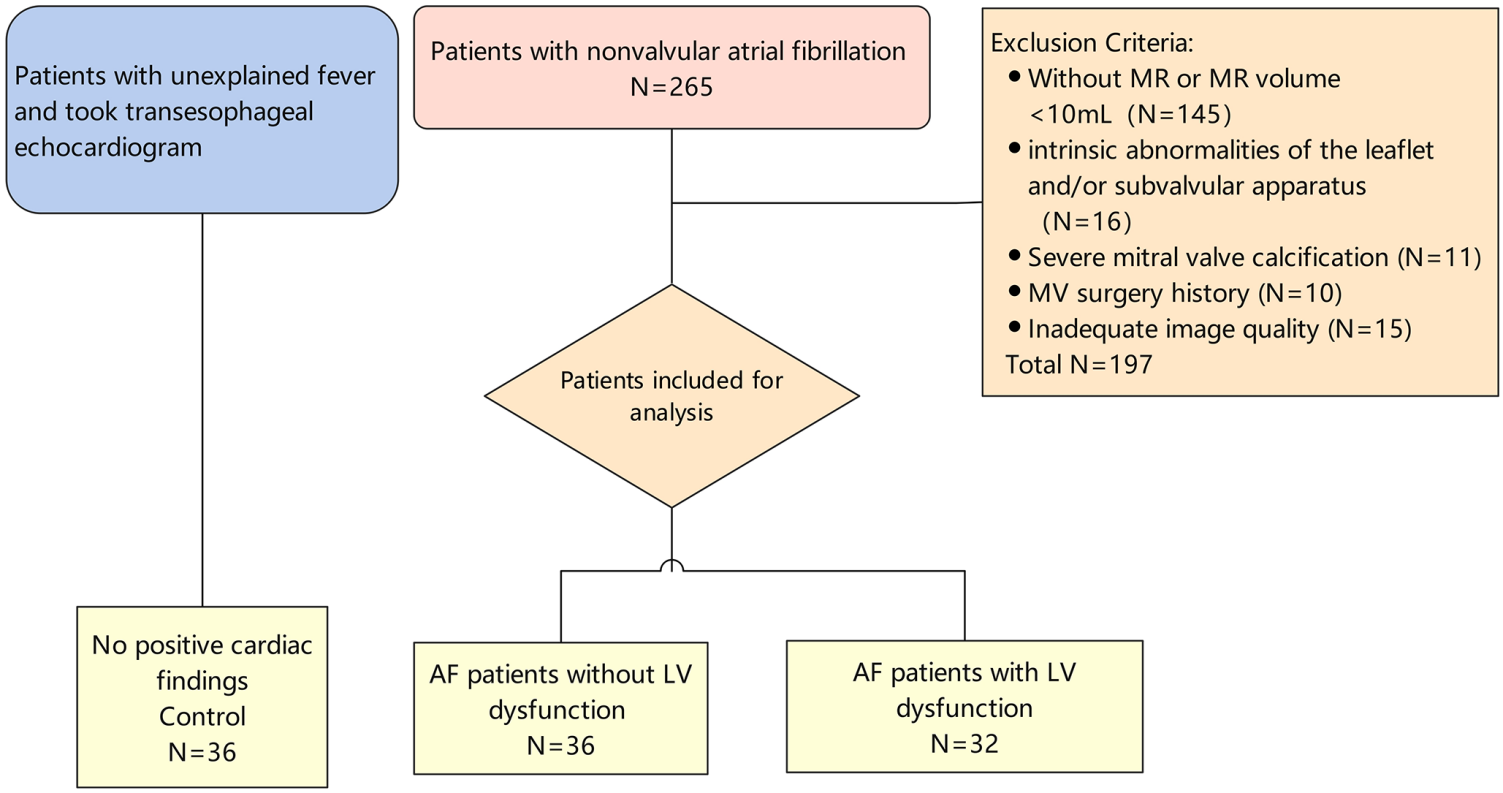
Study flowchart. A total of 104 patients, including 36 with AF without LV dysfunction, 32 with AF and LV dysfunction, and 36 controls, were enrolled

The subjects were enrolled into the present study as illustrated in Fig. 1. FMR was defined as MR without structural MV abnormalities. LV dysfunction was defined as a LVEF ≤ 54% for females and ≤ 52% for males by transthoracic echocardiography (TTE) [8] or as a LV wall motion abnormality. Inclusion criteria were patients diagnosed with AF and without another type of arrhythmia. Patients with the following were excluded: without mitral regurgitation or mitral regurgitation volume < 10 mL, intrinsic abnormalities of the leaflet and/or subvalvular apparatus, severe mitral annular calcification, history of mitral valve surgery, and poor echocardiographic image quality. From April 2020 to April 2022, 68 of 265 nonvalvular AF patients referred for AF catheter ablation or transcatheter MV intervention who underwent TTE and TEE were included. AF patients were divided into two groups. The AFMR group comprised 36 patients with AF, FMR (regurgitation volume ≥ 10 mL), and preserved LV function. The VFMR group comprised 32 patients with AF, FMR (regurgitation volume ≥ 10 mL), and LV dysfunction. In addition, 36 patients with unexplained fever who underwent TEE to screen for infectious endocarditis and without positive findings on echocardiography and electrocardiography were included as the control group. The study protocol was approved by the West China Hospital Ethical Review Committee (approval number: 2020-85).

### Echocardiography

**Fig. 2:**
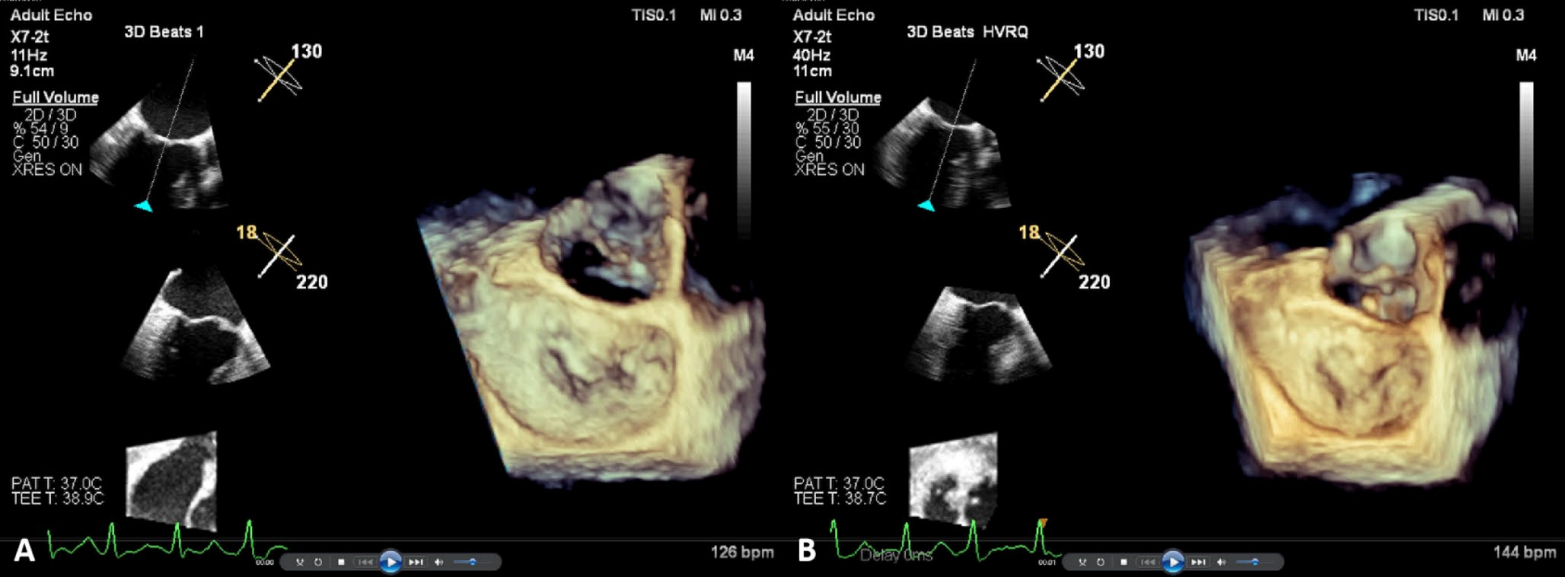
(**A**) One-beat and (**B**) HVR 3D volumes of the MV.

An EPIQ 7 C ultrasound platform (Philips Andover, MA, USA) with the S5-1 probe (frequency: 1–5 MHz) for TTE and the X7-2t probe (frequency: 2–7 MHz) for TEE was used. Routine TTE and TEE were performed by the same proficient physician throughout the study. The modified biplane Simpson method from the apical four- and two-chamber views was used to obtain the LA maximum volume, LA minimum volume, LA ejection fraction (LAEF), LV end-diastolic volume (LVEDV), LV end-systolic volume (LVESV), and LVEF. LVEDV, LVESV, and the LA maximum and minimum volumes were indexed to the body surface area. [8] All patients received local anesthesia before insertion of the TEE probe under the optimal depth and gain conditions. The HVR mode was used to obtain the 3D datasets of the MV, including the annulus, leaflets, and aortic valve. The 3D volumes were recorded at a frame rate of 25–53 volumes per second (Fig. 2). In patients who were in AF rhythm during examination, the volumes were acquired when the heart rate was less than 110 beats per minute.

On the 130–150° MV view, Color Doppler flow imaging was used to determine the presence or absence of MR. In patients with MR, MR was quantified using the PISA method. The effective regurgitant orifice area and regurgitant volume were measured. [9] A regurgitant volume < 10 mL was regarded as trivial MR. The measurements were averaged over five cycles.

### Quantitative assessment of the mitral apparatus

**Fig. 3:**
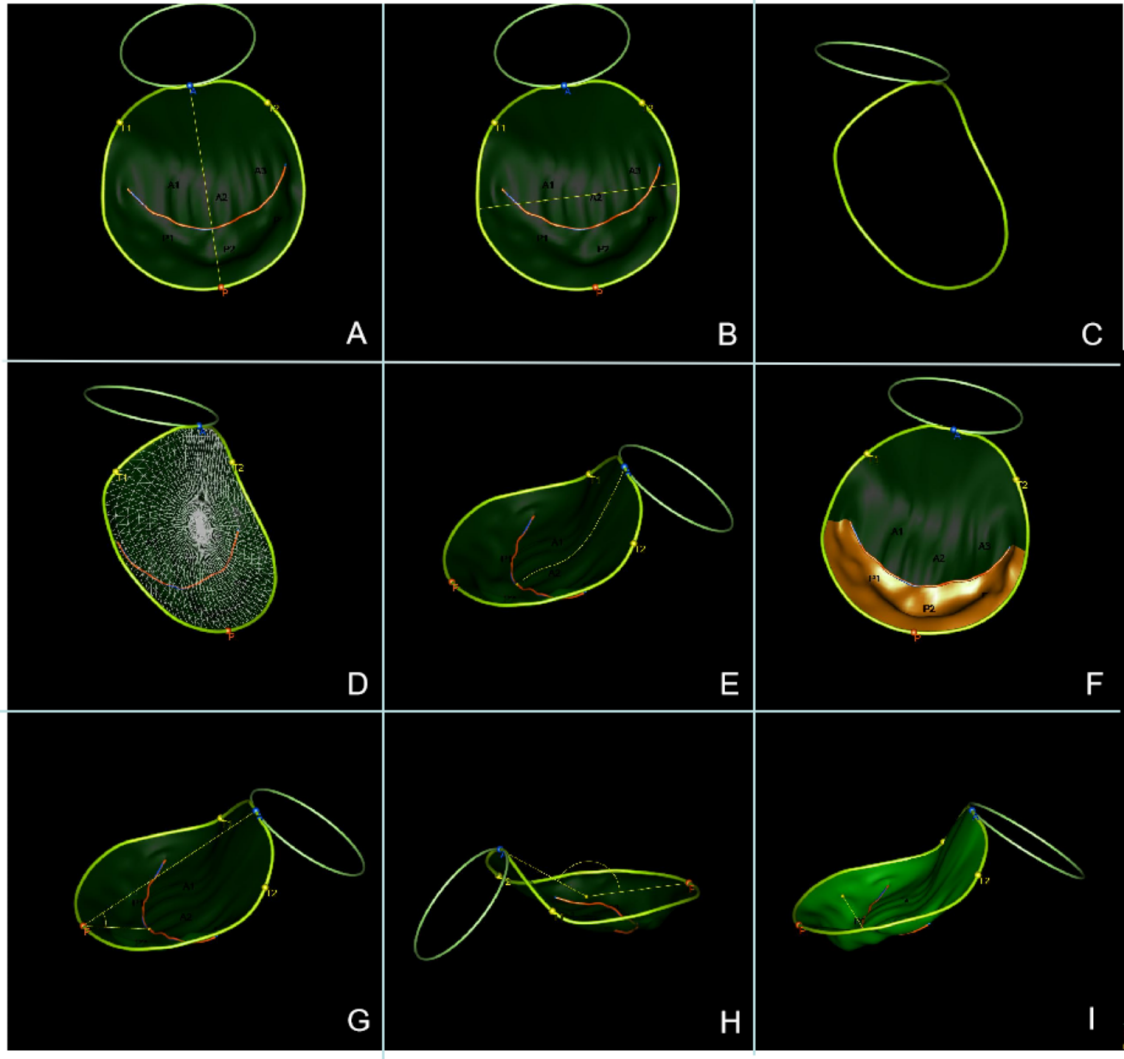
Illustration of the investigated parameters in 3D volume rendered images. (**A**) APD, (**B**) ALPMD, (**C**) SSAP, (**D**) SSAA, (**E**) ALL, (**F**) PLA, (**G**) PML angle, (**H**) NPA, and (**I**) CD.

All echocardiographic images were analyzed offline by a single investigator blinded to the clinical data. Offline analysis of the 3D datasets was performed using the latest QLAB software (version 15; Koninklijke Philips N.V. ADR, MA, USA). In contrast with the previous version, QLAB version 15 can tag the annulus and trace the leaflets automatically, delineating the anterior and posterior leaflets, as well as the coaptation line. This entire procedure requires barely no human intervention. The mitral annulus (MA) includes two regions. One region is located between the left and right fibrous trigones, and is connected anatomically to the aortic annulus at the aortomitral intervalvular fibrous or aortic mitral curtain, which is defined as the intertrigonal annulus. [10] The other region contains the remainder of the annulus, which is classified as the C-shaped annulus. The software automatically generated the following parameters describing the annular geometry (Fig. 3): (1) annulus indices including the anteroposterior diameter of the MA (APD), anterolateral-posteromedial diameter of the MA (ALPMD), sphericity index (SI, the ratio of APD to ALPMD), intertrigonal annulus perimeter (ITAP), C-shaped annulus perimeter (CAP), saddle-shaped annulus perimeter (SSAP), saddle-shaped annulus area (SSAA), annulus height (AH), and non-planar angle (NPA); and (2) leaflet indices: including the anterior leaflet length (ALL), posterior leaflet length (PLL), anterior leaflet area (ALA), posterior leaflet area (PLA), total leaflet area (TLA), distal anterior mitral leaflet (AML) angle, posterior mitral leaflet (PML) angle, coaptation depth (CD), and tenting volume (TV). Each parameter was measured frame by frame, and a parameter-time curve was generated during the whole systole. We observed that the values of most parameters peaked at the start and end of systole, as observed in previous studies; [11] therefore, these two time points were chosen for analyses. Early-systole was defined as immediately after the onset of mitral closure, while end-systole was defined as immediately before aortic closure.

Based on these parameters, dynamic indices were calculated including (1) the ratio of AH to ALPMD = AH/ALPMD, (2) annulus area fraction (AAF)=(SSAA_end−systole_-SSAA_early−systole_)/SSAA_early−systole_, (3) ALA systolic fraction=(ALA_end−systole_-ALA_early−systole_)/ALA_early−systole_, (4) adaptation ratio = TLA/AA, and (5) simplified adaptation ratio=(ALL + PLL)/APD.

### Reproducibility

All geometric parameters of the MV were measured by one practiced physician blind to the patients’ clinical data, and these measurements were compared with the blind measurements of another observer. Intra- and inter-observer variability was evaluated in 20 randomly selected subjects.

### Statistical analysis

Categorical data are presented as frequencies and percentages. After evaluation of normality by the Kolmogorov–Smirnov test, continuous data are presented as mean ± standard deviation or as median (interquartile range). To normalize distributions and improve linearity, the natural log transformation was used for brain natriuretic peptide levels. Group comparisons of baseline characteristics were performed by the chi-squared test for categorical variables and a one-way analysis of variance (ANOVA) with the post-hoc Bonferroni test for continuous variables. The paired Student’s t-test was used to compare the early- and end-systolic values of the same parameters. The linear association between continuous variables was assessed using Pearson’s correlation coefficient. Intraclass correlation coefficients were calculated to assess inter- and intra-observer variability. Statistical testing was two-sided. Results were considered statistically significant at a level of *P* < 0.05. All analyses were performed with the SPSS statistical package version 26.0 (SPSS Inc, Chicago, IL, USA).

## Results

### Clinical characteristics

The general characteristics of the studied patients are summarized in Table 1. The prevalence of hypertension and diabetes mellitus was higher in the AFMR and VFMR groups than in the control group. Patients were older and had higher levels of brain natriuretic peptide in the AFMR and VFMR groups than in the control group. There was no significant difference between the AFMR and VFMR groups, except that the prevalence of coronary atherosclerotic heart disease was significantly higher in the latter group than in the former group.

**Table 1:** Baseline characteristics of the subjects

Characteristics	Control group (n = 36)	AFMR group (n = 36)	VFMR group (n = 32)	P-value*
Age, years	50.8 ± 13.4	66.4 ± 8.5†	62.7 ± 13.7†	< 0.001
Female sex	15/21	17/19	10/22	0.400
Body surface area, m^2^	1.84 ± 0.15	1.80 ± 0.18	1.82 ± 0.17	0.754
Duration of AF, years		2-7.75,3	2–4,3	0.375
AF type (paroxysmal /persistent)		9/27	7/25	0.762
Systolic blood pressure, mmHg	120 ± 10	121 ± 16	126 ± 13	0.157
Diastolic blood pressure, mmHg	79 ± 7	79 ± 10	81 ± 15	0.329
Hypertension	0	22 (61%)	13 (40%)	0.000
Diabetes mellitus, n (%)	2/36	6 (17%)	6 (19%)	0.221
Coronary atherosclerotic heart disease, n (%)		3 (8%)	8 (25%)‡	0.003
BNP (log-transformed)	1.9 ± 0.4	2.9 ± 0.4†	2.9 ± 0.5†	< 0.001
Creatinine, µmol/L	69 (55,74)	83 (69,85)†	85 (73,89)†	0.001
Heart rate, beats/min	89 (75,89)	81 (62,81)†	83 (63,84)†	0.022

Results are shown as the mean ± SD, median (interquartile range), or n (%). *Three and two groups were statistically compared using a one-way ANOVA with post hoc Bonferroni test. Nonnormally distributed continuous variables are presented as median (interquartile range) and compared by means of the non-parametric test. †P < 0.05 vs. control; ‡P < 0.05 vs. AFMR patients. BNP, B-type natriuretic peptide

### Two-dimensional echocardiographic findings

Table 2 shows two-dimensional TTE measurements. The LA diameter and LA maximum volume index were larger in the VFMR and AFMR groups than in the control group. The LV volume was larger in the VFMR group than in the control and AFMR groups. The LVEF was significantly lower in the VFMR group than in the AFMR and control groups. E/e’ was higher in the AFMR and VFMR groups than in the control group. The MR regurgitation volume did not significantly differ between the AFMR and VFMR groups.

**Table 2:** TTE measurements in controls, AFMR group, and VFMR group

	Control group (n = 36)	AFMR group (n = 36)	VFMR group (n = 32)	P-value
LV, mm	46.5 ± 2.5	47.5 ± 5.0	54.4 ± 8†‡	0.001
LA, mm	32.4 ± 4.2	44.6 ± 7.3†	48.4 ± 9.0†	0.001
LA maximum volume, mL	41.0 ± 14.2	103.7 ± 45.3†	120.8 ± 64.2†	0.001
LA maximum volume index, mL/m^2^	22.2 ± 7.3	58.4 ± 27.6†	66.9 ± 34.4†	0.001
LA minimum volume, mL	17.3 ± 8.3	77.0 ± 39.8†	96.0 ± 58.6†	0.001
LAEF, %	59.2 ± 7.1	29.9 ± 8.7†	26.8 ± 8.9†	0.000
LVEDV, mL	100.2 ± 12.9	106.6 ± 27.4	132.2 ± 40.3†‡	0.001
LVESV, mL	33.3 ± 6.5	39.1 ± 13.3	74.5 ± 29.7†‡	0.001
LV EDV/BSA, mL/m^2^	54.3 ± 4.7	59.3 ± 13.9	72.7 ± 22.0†‡	0.001
LV ESV/BSA, mL/m^2^	18.0 ± 3.0	21.7 ± 6.8	40.8 ± 15.8†‡	0.001
LVEF, %	67 ± 4	64 ± 5	43 ± 8†‡	0.001
E/E’ septal	9.9 ± 3	14.9 ± 4.4†	14.6 ± 4.7†	0.001
MR regurgitation volume, mL		18(15,22)	20(15,25)	0.516

Results are shown as the mean ± SD or median (interquartile range). *Three and two groups were statistically compared using a one-way ANOVA with post hoc Bonferroni test. †P < 0.05 vs. control; ‡P < 0.05 vs. AFMR patients. MR, mitral regurgitation

### 3D-TEE analyses

#### MA

MV parameters measured by 3D-TEE are summarized in Table 3. In early- and end-systole, the MA was largest in the VFMR group and smallest in the control group in terms of APD, ALPMD, SSAP, and SSAA. SI in early-systole was significantly higher in the VFMR and AFMR groups than in the control group, indicating that the annulus shape was changed from elliptical to circular in the AFMR and VFMR groups. The NPA was largest in the VFMR group and smallest in the control group, indicating that the saddle-shaped annulus was flattened in the VFMR and AFMR groups. Flattening of the MA was also indicated by analysis of AH/ALPMD, which was lower in the AFMR and VFMR groups than in the control group, but did not significantly differ between the AFMR and VFMR groups.

**Table 3:** 3D-TEE measurements of the mitral annulus/leaflet in the early-systole and end-systole in controls, AFMR group, and VFMR group

	Control group (n = 36)		AFMR group (n = 36)		VFMR group (n = 32)		*P1 for ANOVA	*P2 for ANOVA
	early-systole	end-systole	early-systole	end-systole	early-systole	end-systole	early-systole	end-systole
**Mitral Annulus**								
APD, mm	28.9 ± 3.3	33.4 ± 3.3	35.3 ± 3.8†	37.4 ± 3.5†	37.2 ± 4.5†‡	38.8 ± 4.5†	< 0.001	< 0.001
ALPMD, mm	32.7 ± 3.2	35.4 ± 3.3	37.6 ± 3.2†	38.6 ± 3.3†	39.1 ± 3.4†	39.8 ± 3.4†	< 0.001	< 0.001
SI,%	88.4 ± 7.4	94.5 ± 7.7	93.9 ± 6.6†	97 ± 5.7	95.2 ± 6.6†	97.4 ± 7.2	< 0.001	0.165
CAP, mm	69.9 ± 8.5	75.1 ± 9.2	86.3 ± 6.9†	88.4 ± 7.1†	90.5 ± 8.1†‡	91.9 ± 8.0†	< 0.001	< 0.001
ITAP, mm	34.5 ± 7.1	38.9 ± 7.2	33.5 ± 4.8	35.4 ± 4.9†	35.0 ± 4.6	36.3 ± 4.5	0.559	0.032
SSAP, mm	104.4 ± 9.8	114.0 ± 9.8	119.8 ± 9.8†	123.8 ± 10.0†	125.4 ± 11.8†‡	128.2 ± 11.8†‡	< 0.01	< 0.01
SSAA, cm^2^	8.0 ± 1.5	9.6 ± 1.6	10.8 ± 1.8†	11.6 ± 1.9†	11.9 ± 2.3†	12.4 ± 2.3†	< 0.001	< 0.01
AH, mm	8.6 ± 1.4	8.6 ± 1.4	7.7 ± 1.6	7.8 ± 1.7	7.9 ± 2.2	7.8 ± 2.2	0.051	0.069
NPA, °	140.5 ± 9.1	145.3 ± 6.8	147.2 ± 9.2†	149.7 ± 8.0†	154.1 ± 7.5†‡	155.6 ± 6.8†‡	0.001	0.001
AH/ALPMD, %	26.4 ± 3.5	24.4 ± 3.4	20.5 ± 4.2†	20.2 ± 4.4†	20.1 ± 5.3†	19.5 ± 5.2†	0.001	< 0.001
AAF, %	21.3 ± 6.2		7.2 ± 4.8†		5.0 ± 3.2†		0.001	
**Mitral Leaflets**								
ALA, cm^2^	6.0 ± 1.3	7.0 ± 1.3	7.3 ± 1.4†	7.6 ± 1.4†	8.7 ± 1.8†‡	9.0 ± 1.8†‡	0.001	< 0.001
PLA, cm^2^	3.8 ± 1.1	4.0 ± 1.2	5.2 ± 1.1†	5.2 ± 1.1†	5.9 ± 1.6†‡	5.7 ± 1.6†‡	0.001	0.001
TLA, cm^2^	9.8 ± 1.9	10.9 ± 2.0	12.5 ± 2.1†	12.8 ± 2.1†	14.6 ± 3.0†‡	14.7 ± 3.0†‡	< 0.001	< 0.001
ALL, mm	24.8 ± 3.5	27.1 ± 3.6	27.9 ± 3.1†	28.4 ± 3.1	31.5 ± 4.8†‡	31.9 ± 4.4†‡	< 0.001	< 0.001
PLL, mm	11.7 ± 2.9	11.4 ± 2.8	13.7 ± 1.9†	13.3 ± 1.8†	14.1 ± 2.8†	13.8 ± 3.0†	< 0.001	0.002
Distal AML Angle, °	20.7 ± 4.7	15.4 ± 3.3	18.2 ± 5.3	14.1 ± 4.2	19.2 ± 5.2	16.6 ± 4.8	0.119	0.064
PML Angle, °	51.7 ± 9.3	40.1 ± 8.0	38.4 ± 10.4†	29.9 ± 9.2†	46.5 ± 11.8‡	41.0 ± 11.5‡	< 0.001	< 0.001
TV, cm^3^	2.2 ± 1.0	2.3 ± 1.0	3.1 ± 1.1†	2.5 ± 1.2†	4.7 ± 1.9†‡	4.3 ± 2.0†‡	< 0.001	< 0.001
CD, mm	8.3 ± 1.9	6.8 ± 1.5	8.2 ± 2.4	6.4 ± 2.1	9.7 ± 2.8†‡	8.7 ± 2.8†‡	0.016	< 0.001
ALA/PLA ratio	1.7 ± 0.6	1.9 ± 0.7	1.4 ± 0.3†	1.5 ± 0.3†	1.6 ± 0.4	1.7 ± 0.4	0.011	0.004
ALA fraction	17.5 ± 11.4		4.4 ± 4.7†		3.3 ± 5.1†		0.001	
simplified adaptation ratio	2.2 ± 0.3	1.9 ± 0.2	2.1 ± 0.2	1.9 ± 0.2	2.2 ± 0.3	2.0 ± 0.3	0.382	0.051
adaptation ratio	1.5 ± 0.3	1.4 ± 0.3	1.3 ± 0.1†	1.3 ± 0.1†	1.4 ± 0.1†	1.3 ± 0.1†	< 0.001	< 0.001

* Statistical difference (one-way ANOVA) among the three groups. Data are given as n or mean ± SD. *Statistical difference among the three groups determined using a 1-way ANOVA. Nonnormally distributed continuous variables are presented as median (interquartile range) and compared by means of the non-parametric test. All values except for those of systolic annulus area fraction were measured at early-systole and end-systole. †P < 0.05 vs. Control group, ‡P < 0.05 vs. AFMR group. APD, anteroposterior diameter of mitral annulus; ALPMD, anterolateral-posteromedial diameter of mitral annulus; SI, sphericity index; ITAP, Intertrigonal annulus perimeter; CAP, C-Shaped Annulus Perimeter; SSAP, saddle shaped annulus perimeter; SSAA, saddle shaped annulus area; AH, annulus height; NPA, non-planar angle; AAF, annulus area fraction; ALL, anterior leaflet length; PLL, posterior leaflet length; ALA, anterior leaflet area; PLA, posterior leaflet area; TLA, total leaflet area; AML, anterior mitral leaflet; PML angle, posterior mitral leaflet; CD, coaptation depth; TV, tenting volume

The two parts of the MA exhibited different variations in the AFMR and VFMR groups. The ITAP did not significantly differ among the three groups, whereas the CAP was significantly larger in the VFMR and AFMR groups than in the control group. This result confirms that the C-shaped MA is more easily expanded than the intertrigonal annulus in AFMR and VFMR patients.

During ventricular contraction, there is progressive annular enlargement from early to late systole. The AAF, which reflects MV annular deformability in systole, was significantly lower in the AFMR and VFMR groups than in the control group.

### Mitral leaflets

The ALA and PLA were slightly larger in the AFMR group than in the control group, and were greatly enlarged in the VFMR group. The ALL and PLL exhibited a similar trend. The increases of the ALA and PLA in the AFMR and VFMR groups reflect leaflet adaptative growth secondary to MA dilation, whereas the decrease of the ALA/PLA ratio in these groups demonstrate the leaflet growth percentage of the AML less than the PML. The adaptation ratio was lower in the AFMR and VFMR groups than in the control group, indicative of insufficient leaflet adaptative growth. However, the simplified adaptation ratio did not significantly differ among the three groups. These results demonstrate that the simplified adaptation ratio is insensitive to evaluate leaflet adaptative growth compared with the adaptation ratio.

The ALA gradually increased from early- to end-systole in all groups, whereas the PLA did not significantly vary in any group. The ALA systolic fraction was significantly decreased in the VFMR and AFMR groups. This is thought to reflect a chain reaction caused by reduced contractility of the MA.

The PML angle was smallest in the AFMR group and largest in the control group, whereas the distal AML angle did not significantly differ among the three groups. Accordingly, the CD was significantly increased in the VFMR group, but did not significantly differ between the AFMR and control groups. The TV was largest in the VFMR group and smallest in the control group. However, the timing of the peak TV was irregular and differed between individuals, which hampered intergroup comparisons of this parameter.

### Correlation analysis

**Fig. 4:**
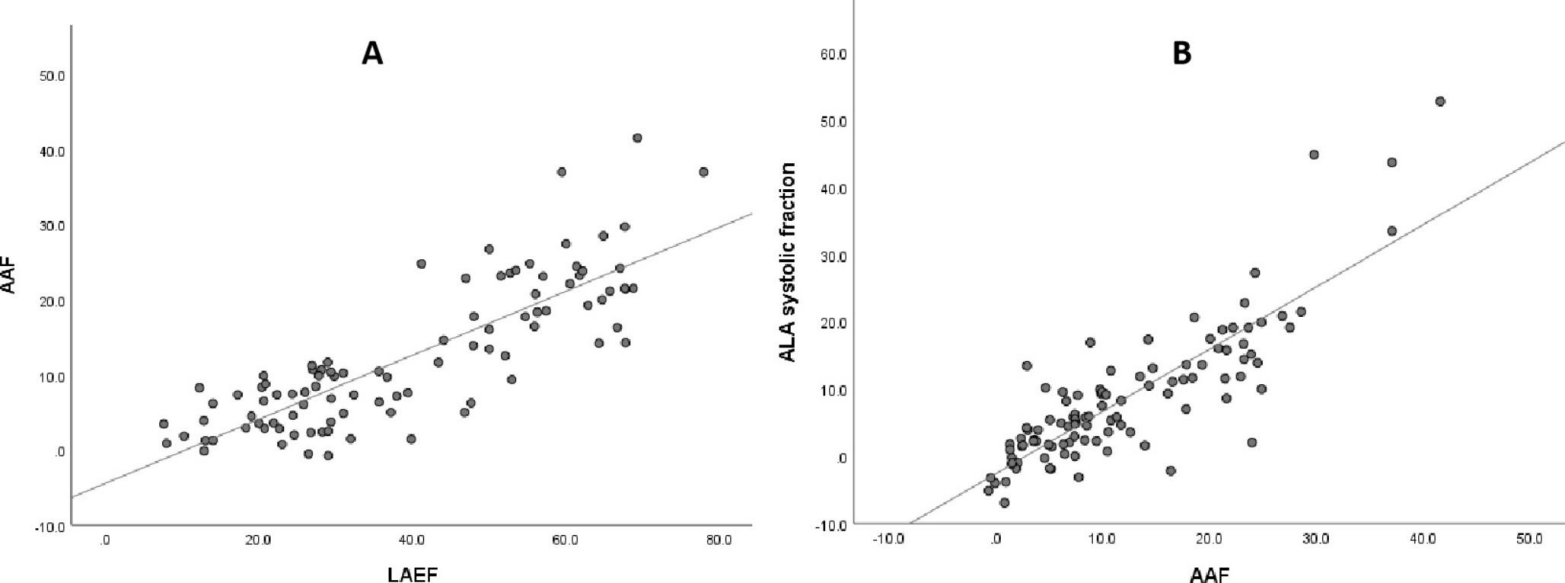
(**A**) A scatter plot of the mitral AAF and LAEF (R^2^ = 0.673). (**B**) A scatter plot of the mitral AAF and AML area fraction (R^2^ = 0.719). The dotted line represents the line of best fit

In univariate analysis, the AAF was significantly correlated with the LAEF (r = 0.830, P < 0.001) and ALA systolic fraction (r = 0.848, P < 0.001) (Figure 4). There was no significant correlation between the MR regurgitation volume and other parameters. There was a weak correlation between NPA and AH/ALPMD (r=-0.56 and r=-0.58 in early- and end-systole, respectively).

### Reproducibility of quantitative 3D analysis

Intraclass correlation coefficients were obtained for MV parameters. Excellent reproducibility was observed in both inter- and intro-observer analyses (Table 4).

**Table 4:** Strength of agreement in intra- and interobserver analyses

	Intraclass correlation coefficients (95%CI)	
	Intraobserver	Interobserver
APD, mm	0.96 (0.90–0.98)	0.92 (0.81–0.97)
ALPMD, mm	0.97 (0.918–0.987)	0.92 (0.81–0.97)
SSAP, mm	0.96 (0.90–0.98)	0.95 (0.89–0.98)
SSAA, cm^2^	0.96 (0.90–0.98)	0.95 (0.89–0.98)
NPA, °	0.93 (0.84–0.97)	0.76 (0.50–0.90)
ALA, cm^2^	0.95 (0.85–0.98)	0.75 (0.50–0.90)
PLA, cm^2^	0.94 (0.84–0.98)	0.76 (0.51–0.91)
TV, mL	0.97 (0.92–0.99)	0.96 (0.90–0.98)
CD, mm	0.93 (0.88–0.97)	0.88 (0.72–0.95)

APD, anteroposterior diameter of mitral annulus; ALPMD, anterolateral-posteromedial diameter of mitral annulus; SSAP, saddle shaped annulus perimeter; SSAA, saddle shaped annulus area; NPA: nonplanar angle; ALA, anterior leaflet area; PLA, posterior leaflet area; TV, tenting volume; CD, coaptation depth

## Discussion

Using HVR 3D-TEE, the present study characterized the detailed mitral geometric changes in patients suffering from AF with and without LV dysfunction and compared them with controls. In AF patients, (1) multiple factors are linked with atrial FMR including MA dilatation, a flattened annular saddle shape, reduced annular contractility, and PML tethering; (2) LA remodeling leads to morphological and functional changes of the C-shaped MA and ML growth; and (3) atriogenic PML tethering decreases the PML angle, which is the most influential factor in atrial FMR.

Very few studies have evaluated the mechanism underlying atrial FMR based on HVR 3D-TEE [11]. In our study, we successfully used HVR 3D-TEE, whereas most previous studies used one-beat 3D-TEE. The temporal resolution of HVR 3D-TEE is 2–4-fold higher than that of one-beat 3D-TEE, which increases the likelihood of discerning rapid movements and thus improves the accuracy of quantification. Moreover, the image quality of HVR 3D-TEE is good enough to ensure the accuracy of quantification due to good acoustic transmission in transesophageal examination.

The MA includes two regions, namely, the intertrigonal annulus, which has a stable and fixed position in the heart, and the C-shaped annulus, which is a discontinuous fibrous arc attached to the junction of the left atrium and free wall of the left ventricle. [10, 12, 13] In our study, the intertrigonal annulus was stable in the different groups. By contrast, the C-shaped annulus was significantly enlarged in AFMR and VFMR patients, suggesting that it was easily stretched outward upon LA remodeling. This finding is consistent with a previous report. [14] AF leads to enlargement and flattening of the mitral ring, which is the basis of atrial MR occurrence. The non-planar saddle shape of the MA helps to maintain normal valve function by decreasing the blood flow pressure on the valves. [15, 16] A previous study reported that the NPA is better than AH/ALPMD at reflecting flattening of the MA because it is independent of changes in the pre- and afterload heart. In addition, the nadir point of AH is the midpoint of the posterior mitral valve annulus, and the nadir point of the NPA is the midpoint of the ALPMD, which means these two parameters are not uniform, and there is only a weak correlation between the NPA and AH/ALPMD. [15] In our study, the increased NPA confirmed that the MA was flattened in AF patients with and without LV dysfunction. The MA was more flattened in the VFMR group than in the AFMR group.

The MA does not actively contract by itself, but moves passively upon LA and LV contraction. AF causes microscopic and macroscopic LA remodeling, as well as simultaneous electrical alteration. [17, 18] Interstitial fibrosis leads to gradually replacement of the contractile atrial myocardium. In our study, the left atrium was significantly larger and the LAEF was significantly lower in the AFMR and VFMR groups than in the control group, and the LAEF was positively correlated with the AAF. All these findings prove that LA remodeling is responsible for reduced MA contractility. Appropriate annular motion is required for successful leaflet coaptation, and reduced annular dynamics is an important cause of atrial FMR.

It was previously reported that mitral leaflet active adaptive growth compensates for LV remodeling and that leaflet growth is triggered by a complex signaling cascade, a process called the endothelial-mesenchymal transformation. [19] In atrial FMR, leaflet enlargement was also reported to be a compensatory mechanism for the dilated MA and tethering of the MV due to LV dilatation. [6, 20] In our study, the mitral leaflet was enlarged in AF patients consistent with previous studies. We further found that the PML grew significantly more than the AML. In addition, the adaptation ratio was lower in the AFMR and VFMR groups than in the control group, supporting the view that leaflet growth is insufficient relative to annular enlargement in AFMR patients. [21] However, in our study, the leaflet area was measured in systole, during which the MV is closed, and was underestimated because the measurement did not include the coaptation area. This may have affected the accuracy of the results. Therefore, leaflet remodeling in patients with atrial FMR remains to be further investigated.

In recent years, some studies have pointed out that more focus is required on PML tethering in AF patients. [22, 23] The PML attachment is pressed against the crest of the LV inlet, and traction of the LA wall causes displacement of the posterior annulus onto the crest of the LV inlet, causing tethering of the PML. In our study, the distal AML angle was stable in the different groups, whereas the PML angle was decreased in the AFMR group. These findings support the view that expansion of the LA wall leads to deviation of the posterior annulus toward the outside of the myocardium, causing the PML angle to decrease. Our findings are consistent with those of Ito et al. and Nakai et al. [24, 25] However, another study reported that the PML angle is increased in patients with AF. [21] The difference in results may be due to use of different study populations and types of analysis software.

In this study, enlargement and flattening of the mitral ring were exacerbated in AF patients with LV dysfunction. However, MR in AF patients with LV dysfunction was not worse than in AF patients without LV dysfunction. By comparing AF patients with and without ventricular dysfunction, except for PML angle, other 3D-TEE measurements of the mitral valve showed the same trend in the VFMR group and the AFMR group, or more significant in the VFMR group, suggesting atriogenic PML tethering is probably the most influential factor in atrial FMR. Atriogenic tethering of the PML, complicated by MA dilatation, further decreases leaflet coaptation, thereby exacerbating atrial FMR.

### Study limitations

First, this was a single-center study, and the number of patients included was relatively small. We could not separate patients with mild, moderate, and severe atrial MR for grade comparisons. We measured the regurgitant volume and found no significant difference between AF patients with and without ventricular dysfunction. Hence, we believe that integrated analysis of MR is applicable to clinical practice. Second, the 3D datasets were acquired in a HVR mode in patients with an AF rhythm during echocardiography. We chose a cycle when the rhythm variation was relatively stable and the heart rate was slow, and the measurements were not averaged. However, inter- and intra-observer variability of MV measurements demonstrated a good level of reproducibility. Hence, we believe the quantification was accurate. Third, in the VFMR group, AF occurred after LV dysfunction; therefore, LA remodeling was mainly caused by LV dysfunction. Further investigation of a larger number of FMR patients with LV dysfunction and without AF is desirable to confirm the results of this study. Finally, the controls were recruited from patients referred for clinically indicated TEE examination; therefore, they are not a true sample of the general population, and the age of patients could not be matched. Most AF patients were taking β-blockers to lower their heart rates; therefore, heart rates differed between the AF groups and control group. However, it is not justifiable to recruit normal volunteers for TEE examination considering the risks associated with intubation.

## Conclusions

Although HVR has been available on Philips ultrasound equipment for more than 10 years, it is mainly used to quantify the heart chamber volume. [26, 27] This study found that HVR 3D-TEE has acceptable image quality and improved temporal resolution, and is especially suitable for patients with arrhythmia or tachycardia. This will likely facilitate the widespread implementation of 3D-TEE for MV assessment in clinical practice.

There are multiple reasons for atrial FMR. In our study, LA remodeling caused expansion of the MA and reduced mitral contractility, disruption of the saddle shape of the MA, and PML tethering. All these factors, especially atriogenic PML tethering, are responsible for atrial FMR occurrence. In addition, inadequate adaptative growth of the mitral leaflets should be considered. Therapeutic strategies to prevent or reverse LA remodeling caused by AF should be emphasized to prevent the development of atrial FMR. [28, 29]
